# Dynamics of macrophage populations of the liver after subtotal hepatectomy in rats

**DOI:** 10.1186/s12865-018-0260-1

**Published:** 2018-07-09

**Authors:** Andrey V. Elchaninov, Timur Kh. Fatkhudinov, Natalia Y. Usman, Evgeniya Y. Kananykhina, Irina V. Arutyunyan, Andrey V. Makarov, Anastasia V. Lokhonina, Irina Z. Eremina, Viktor V. Surovtsev, Dmitry V. Goldshtein, Galina B. Bolshakova, Valeria V. Glinkina, Gennady T. Sukhikh

**Affiliations:** 1 National Medical Research Center for Obstetrics, Gynecology and Perinatology named after Academician V.I.Kulakov of Ministry of Healthcare of Russian Federation, 4 Oparina Street, 117997 Moscow, Russia; 2 Scientific Research Institute of Human Morphology, 3 Tsurupa Street, 117418 Moscow, Russia; 3 Ministry of Healthcare of the Russian Federation, Pirogov Russian National Research Medical University, 1 Ostrovitianov Street, 117997 Moscow, Russia; 4 Peoples Friendship University of Russia (RUDN University), 6 Miklukho-Maklaya Street, 117198 Moscow, Russia; 5 Research Center of Medical Genetics, 1 Moscvorechie, 115478 Moscow, Russia

**Keywords:** Liver, Regeneration, Kupffer cells

## Abstract

**Background:**

In many clinical cases of extensive liver resection (e.g. due to malignancy), the residual portion is too small to maintain the body homeostasis. The resulting acute liver failure is associated with the compensatory growth inhibition, which is a typical manifestation of the ‘small for size’ liver syndrome. The study investigates possible causes of the delayed onset of hepatocyte proliferation after subtotal hepatectomy (80% liver resection) in rats.

**Results:**

The data indicate that the growth inhibition correlates with delayed upregulation of the *Tnf* gene expression and low content of the corresponding Tnfα protein within the residual hepatic tissue. Considering the involvement of *Tnf*/Tnfα, the observed growth inhibition may be related to particular properties of liver macrophages – the resident Kupffer cells with CD68^+^CX1CR3^−^CD11b^−^ phenotype.

**Conclusions:**

The delayed onset of hepatocyte proliferation correlates with low levels of Tnfα in the residual hepatic tissue. The observed growth inhibition possibly reflects specific composition of macrophage population of the liver. It is entirely composed of embryonically-derived Kupffer cells, which express the ‘proregeneratory’ M2 macrophage-specific marker CD206 in the course of regeneration.

**Electronic supplementary material:**

The online version of this article (10.1186/s12865-018-0260-1) contains supplementary material, which is available to authorized users.

## Background

Macrophages are known to participate in the coordination of tissue regeneration. They constitute a heterogeneous category of cells, which differ in their origin and functional properties [1].

Macrophages originate from three different hemopoietic cell sources: yolk sac, embryonic/fetal liver, and red bone marrow. In many adult organs, macrophages are a mixture of descendants from both the embryonic liver and the red bone marrow (whether hemopoietic stem cells of the yolk sac are also involved remains as yet unexplored) [1]. By contrast, the central nervous system and the liver comprise their own specific macrophage populations, which originate almost exclusively from the hemopoietic cells of embryonic liver (whereas, for example, macrophages of the dermis or the intestinal mucosa are represented predominantly by cells of monocytic origin). Source-specific contributions of macrophages to normal function and regeneration of different organs remain obscure.

In addition to the multiple sources of origin, activated macrophages may differ by their functional properties. In particular, the classically-activated M1 macrophages, which secrete pro-inflammatory Il1b, Il6, and Tnfα, are opposed to the alternatively-activated M2 macrophages, which support regeneration; M2 macrophages secrete anti-inflammatory Il10 [2]. There is still no certainty about correlation of the source of origin of a macrophage with its functional type [1].

In non-regenerating healthy liver, the absolute majority of macrophages are resident Kupffer cells, and quite a few of them descend from blood monocytes [3, 4]. All macrophages of monocytic origin express specific surface antigens (Cx3cr1, CD11b, and the mouse-specific Ly6C), which are missing in Kupffer cells [3, 4]. Differential roles of monocyte-derived macrophages and Kupffer cells in liver regeneration remain elusive. However, it has been shown that both the depletion of Kupffer cells and the block of infiltration with blood monocytes slow down the regeneration [4, 5].

In many clinical cases of extensive liver resection (e.g. due to malignancy), the residual portion is too small to maintain the body homeostasis. [6]. The resulting acute liver failure is associated with the compensatory growth inhibition, which is a typical manifestation of the ‘small for size’ liver syndrome. Mechanisms of this inhibition (frequently resulting in a complete block of regeneration and death) remain understudied.

Subtotal hepatectomy (SH) in rodents, which is the resection of 80% of the liver mass, may be considered as a model of the ‘small for size’ liver syndrome. Experiments with the compensatory growth of liver tissue in rats after SH show prolonged arrest of hepatocyte proliferation [7], molecular mechanisms of which are still obscure [8, 9].

The study investigates the causes of the delayed onset of hepatocyte proliferation after SH in rats. Particular attention is focused on the possible role of macrophages in this delay.

## Methods

### Model

The outbred eight-week-old male Sprague-Dawley rats of 250–300 g weight were obtained from the Institute for Bioorganic Chemistry branch animal facilities (Pushchino, Moscow region, Russia). All experimental work involving animals was carried out according to the standards of laboratory practice (National Guidelines No. 267 by Ministry of Healthcare of the Russian Federation, June 1, 2003), and all efforts were made to minimize the suffering. The study was approved by the Ethical Review Board at the Scientific Research Institute of Human Morphology (Protocol No. 5, March 12, 2013).

The animals were operated between 9 am and 11 am under general anesthesia with diethyl ether (Medhimprom, Moscow region, Russia; 0.08 ml per liter of chamber volume [10]). The surgery was performed as described elsewhere [11, 12]. The operated animals, two per cage, were housed for recovery in a room with controlled temperature, 12:12 h light-dark cycle, and unlimited access to standard food and water. The animals were drawn from the experiment in CO_2_-chamber at 24 h, 30 h, 48 h, 72 h, 5 days, 7 days, or 10 days after the surgery (5–6 animals for each term).

Borderline condition produced by subtotal 80% hepatectomy in a rat is similar to acute liver failure. The condition is resolved in the course of 48 h by either spontaneous death, or switch to recovery, and no means for distinction between the survivors and non-survivors have been reported. This is the reason for the non-use of euthanasia in this study [11, 12].

The regenerating livers were promptly dissected, weighed, and preserved for analysis. Hepatic tissue from non-operated or sham-operated animals was utilized as an additional control material for the assessments of liver mass recovery and hepatocyte proliferation, functional tests, immunostaining, and western-blot analysis. The sham operation procedure exactly reproduced all steps of the surgery, but the liver lobes were only briefly externalized and then returned into their original position. The non-operated control group included intact male rats (*n* = 10) matching all parameters of the experimental group.

Residual livers grew to the initial liver volume by day 10 after the surgery. By this time, serum albumin concentrations and ALT activity returned to their original values indicating functional recovery [12].

### Hepatocyte proliferation assay

Hepatocyte proliferation was evaluated by differential counts of Ki67 positive and negative cells in immunostained cryosections, with a total of 3 × 10^3^ cells assessed for each animal.

### Fluorescence microscopy

The tissues were preserved in liquid nitrogen, and 5–7 μm cryosections were prepared. Immunostained cryosections were analyzed using Leica DM4000 B fluorescence microscope (Leica Microsystems CMS GmbH, Wetzlar, Germany).

### Immunostaining of macrophages

The total macrophage population was specifically stained with anti-CD68 antibody (Abcam, Cambridge, UK) in cryosections, whereas the М2 macrophages were selectively identified by anti-CD206 immunostaining (Santa Cruz Biotechnology, Dallas, TX, USA). After incubation with FITC-conjugated secondary antibodies (Abcam, Cambridge, UK) and the signal development, cell nuclei were counterstained with DAPI (Sigma-Aldrich, St. Louis, MO, USA). Positive and negative cells were counted to calculate corresponding indexes, with a total of 3 × 10^3^ cells assessed for each animal.

Macrophages derived from blood monocytes were selectively identified by anti-CX3CR1 immunostaining (Abcam, Cambridge, UK) and anti-CD11b immunostaining (Santa-Cruz, USA) in formalin-fixed paraffin-embedded hepatic tissue sections, in comparison with the splenic tissue sections for a positive control. Primary antibodies were applied in 1:100 dilutions, after pretreatment of the slides with sodium citrate buffer (10 mM sodium citrate, 0.05% Tween 20, pH 6.0) pre-heated in accordance with the epitope retrieval protocol recommended by the manufacturer. Immunoperoxidase-stained sections were counterstained with hematoxylin; the positive and negative cells were counted to calculate corresponding indexes, with a total of 3 × 10^3^ cells assessed for each animal.

### Mitotic index evaluation

Macrophages, mitotically inactive and dividing, > 1000 cells per animal, were differentially counted in the formalin-fixed paraffin-embedded, anti-CD68/immunoperoxidase stained sections. The counts for non-operated rats of similar age (*n* = 10) were used for comparison.

### Western-blotting

Proteins, isolated from the liquid nitrogen-preserved liver tissue using MicroRotofor™ Cell Lysis Kit, were quantified by Bradford assay using Quick Start™ Bovine γ-Globulin Standard. The protein extracts were mixed with 4× Laemmli Sample Buffer (Bio-Rad, Hercules, CA, USA) (1,1) and heated at 95 °C for 5 min immediately before loading. The proteins were separated by 10% sodium dodecyl sulfate polyacrylamide gel electrophoresis (SDS-PAGE), and transferred to PVDF membrane (Bio-Rad, Hercules, CA, USA). Tnfα levels were evaluated by Western-blot analysis using Trans-Blot® Turbo™ RTA Mini LF PVDF Transfer Kit Bio-Rad Laboratories, Inc. (Hercules, CA, USA) for protein transfer. Other Bio-Rad products used in the assay include Immun-Star Goat Anti-Rabbit (GAR)-HRP Conjugate as secondary antibodies, Clarity™ Western ECL with ChemiDoc™ system for signal development, and Image Lab™ software for data analysis.

The membranes were blocked with 5% milk in Tris-buffered saline with 0.1% Tween 20 (TTBS) for 1 h at room temperature and subsequently incubated with primary antibodies to Tnfα or β-tubulin (Abcam, Cambridge, UK) applied in 1:100 dilutions, as recommended by the manufacturer. The full-length images of blots are included in a Supplementary Information file. All blots were processed in parallel.

### Statistical analysis

The data were analyzed using SigmaStat 3.5 (Systat Software Inc., Chicago, IL, USA). Sample proportions were compared by 2-sample z-test; more-than-two-groups comparisons were done using ANOVA on ranks; *p* < 0.05 for the differences were considered statistically significant.

## Results

### Ki67 protein expression dynamics

**Fig. 1:**
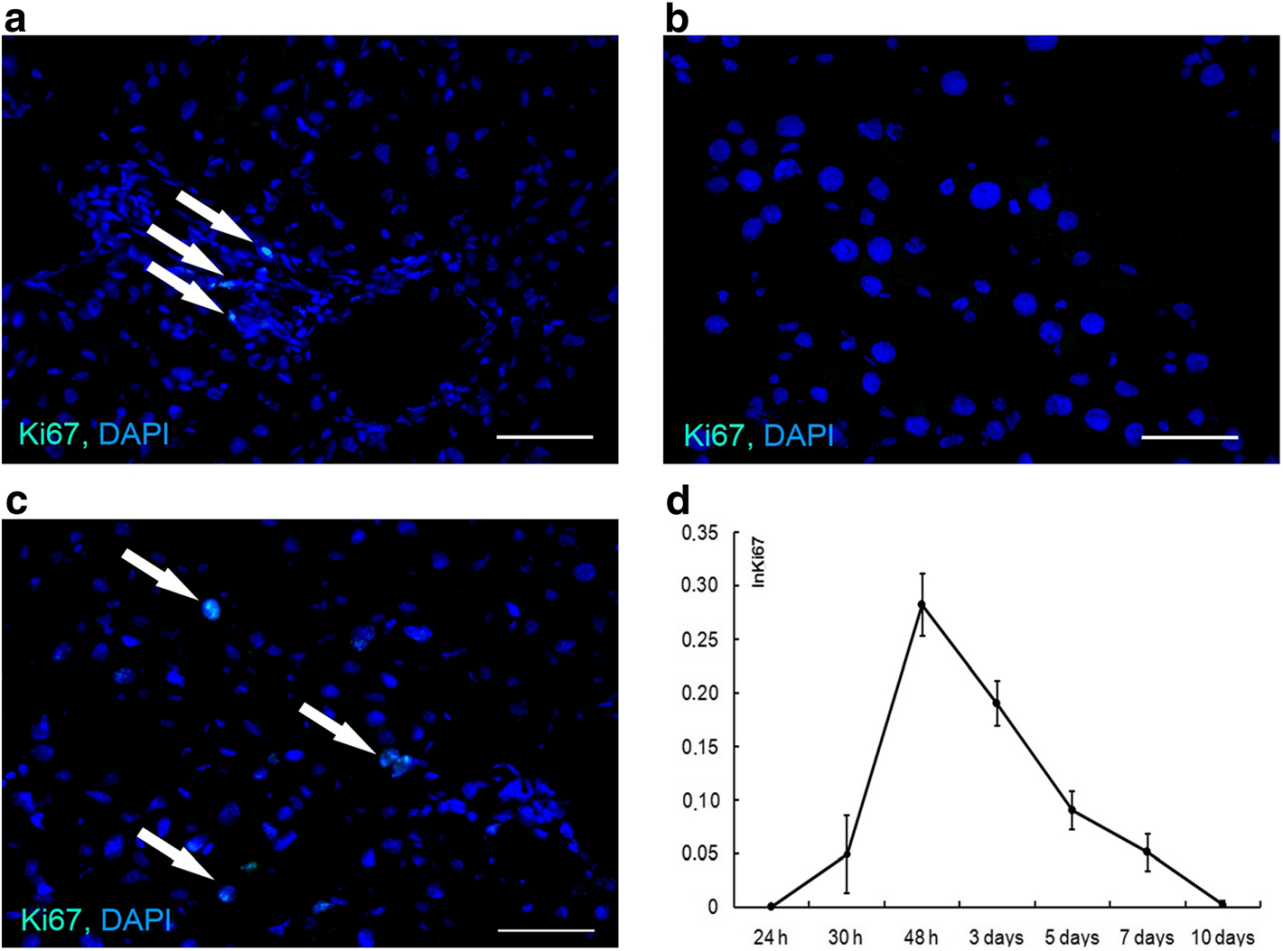
Hepatocyte proliferation dynamics. Ki67 expression in the intact liver (**a**) and in the residual livers at 24 h (**b**) and at 30 h (**c**) after the surgery. Index of Ki67^+^ hepatocytes is plotted against time after the surgery (**d**). The data are represented as mean values ± SD. Bars, 50 μm; cell nuclei are counterstained DAPI (blue). Arrowheads indicate Ki67^+^ cells (**a**, **b**, **c**)

Solitary Ki67^+^ cells were observed in the livers of intact and sham-operated animals, mostly in the vicinity of triads. Judging by their size and morphology, these cells could not be hepatocytes (Fig. 1a). The first Ki67^+^ hepatocytes emerged at 30 h after the surgery, their numbers reached maximum at 48 h after the surgery, and decreased after that point (Fig. 1b, c, and d).

### Liver macrophage population dynamics

**Fig. 2:**
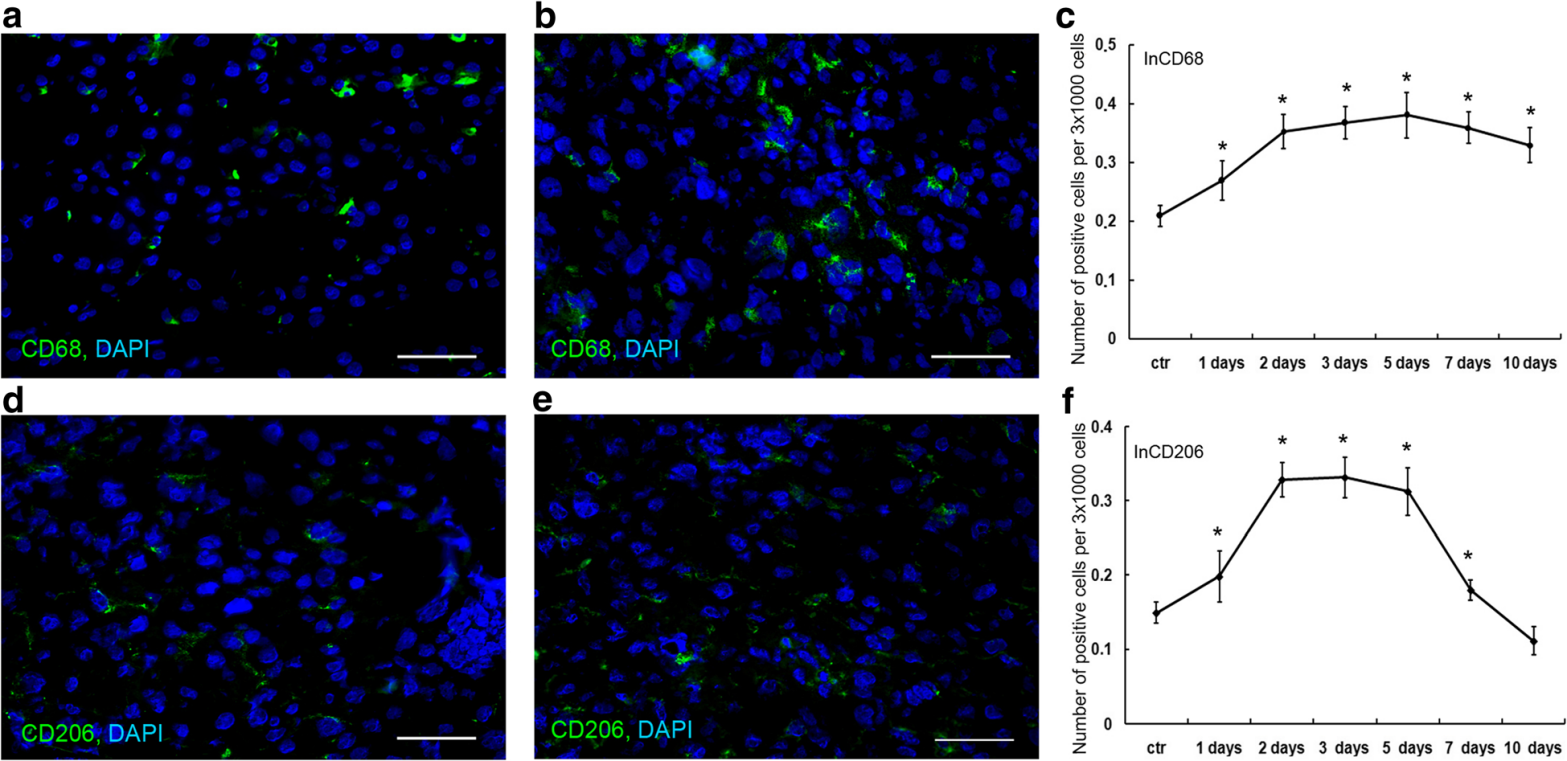
Immunostaining and quantification of CD68^+^ and CD206^+^ macrophages in the residual liver. CD68^+^ cells (green) in the intact liver (**a**) and in the residual liver on day 3 after the surgery (**b**). The diagram shows dynamic changes in the CD68^+^ cell content in the course of regeneration, index of CD68^+^ content (InCD68) (**c**). Relative quantities of CD206^+^ cells (green) in the intact liver (**d**) and in the residual liver on day 3 after the surgery (**e**). The diagram shows dynamic changes in the CD206^+^ cell content in the course of regeneration, index of CD206^+^ content (InCD206) (**f**). The data are represented as mean values ± SD with the asterisks indicating statistical significance of the differences (as compared with the control; *p*˂0.05). Bars, 50 μm; cell nuclei are counterstained with DAPI (blue)

In accordance with the statement that macrophages constitute a numerous cell population in the intact liver, approx. 20% of cell nuclei observed in the sections of intact liver belonged to CD68^+^ cells (Fig. 2a).

CD68^+^ cells significantly increased in number by day 1 after the surgery, and their numbers remained increased, as compared with the control, until the end of observation (*p* < 0.001, Fig. 2b and c).

Alternatively activated anti-inflammatory M2 macrophages (defined as CD206^+^ cells), almost totally missing in the intact livers (Fig. 2d, and f), dramatically increased in number by day 1 after the surgery (*p* < 0.05, Fig. 2e). These cells, found predominantly in the vicinity of liver sinusoids, were the most numerous on days 2 to 5 after the surgery. Their numbers started to decrease later on and returned to the initial level by day 10 after the surgery (*p* > 0.05, Fig. 2f).

**Fig. 3:**
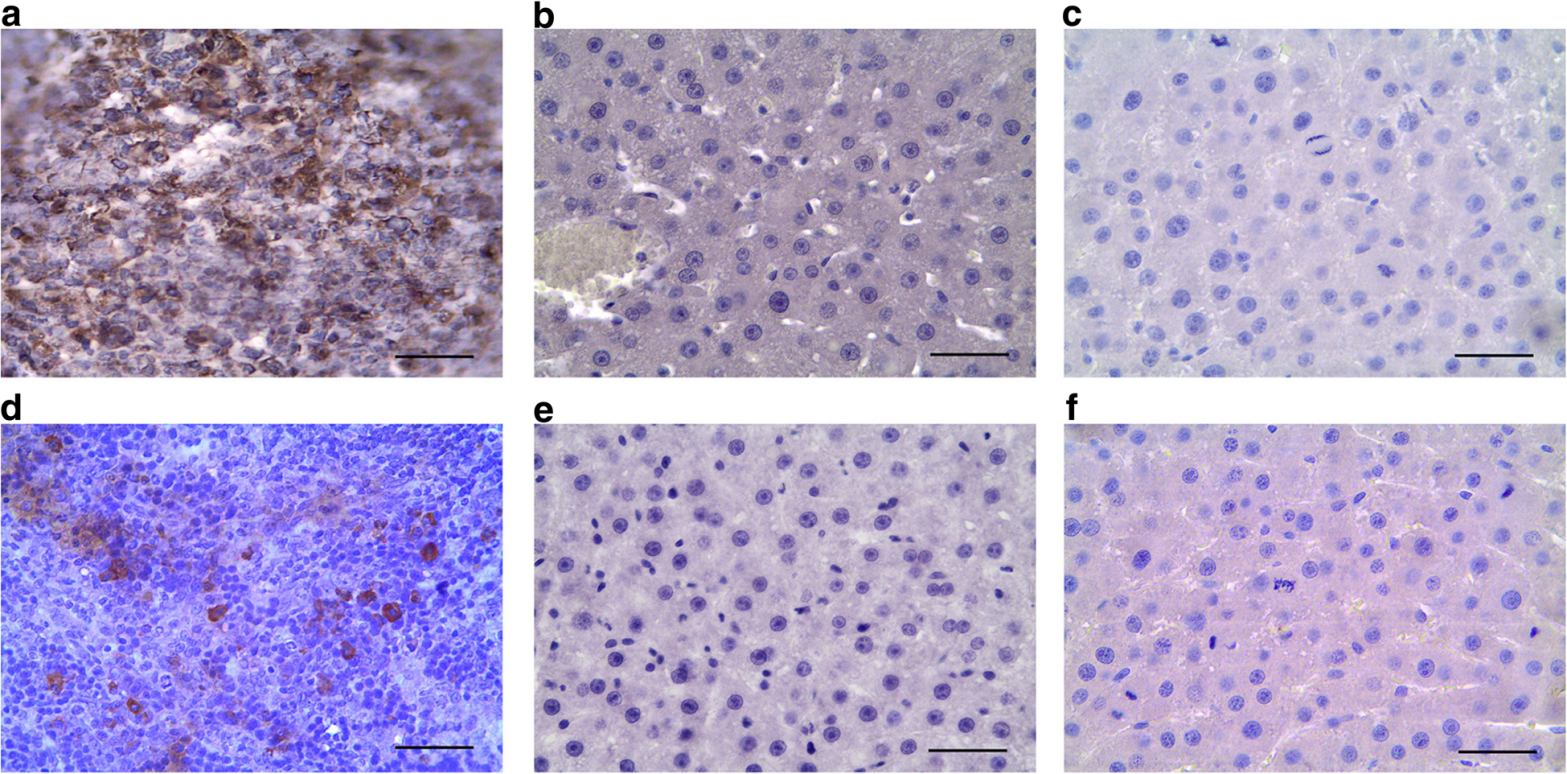
Immunostaining of CX3CR1^+^ and CD11b^+^ cells in splenic and hepatic tissues. Expression of CX3CR1 in intact spleen (positive control, **a**). Expression of CX3CR1 in the intact liver (**b**) and in the residual liver tissue on day 3 after the surgery (**c**). Expression of CD11b in intact spleen (positive control, d). Expression of CD11b in the intact liver (**e**) and in the residual liver tissue on day 3 after the surgery (**f**). Bars, 50 μm; cell nuclei are counterstained with hematoxylin (blue)

All CD68^+^ cells observed in this study, in both intact and regenerating livers, were Kupffer cells, because none of them were positive for Cx3cr1 or CD11b surface antigens specific to the monocyte-derived macrophages (Fig. 3).

### Liver macrophage proliferation dynamics

**Fig. 4:**
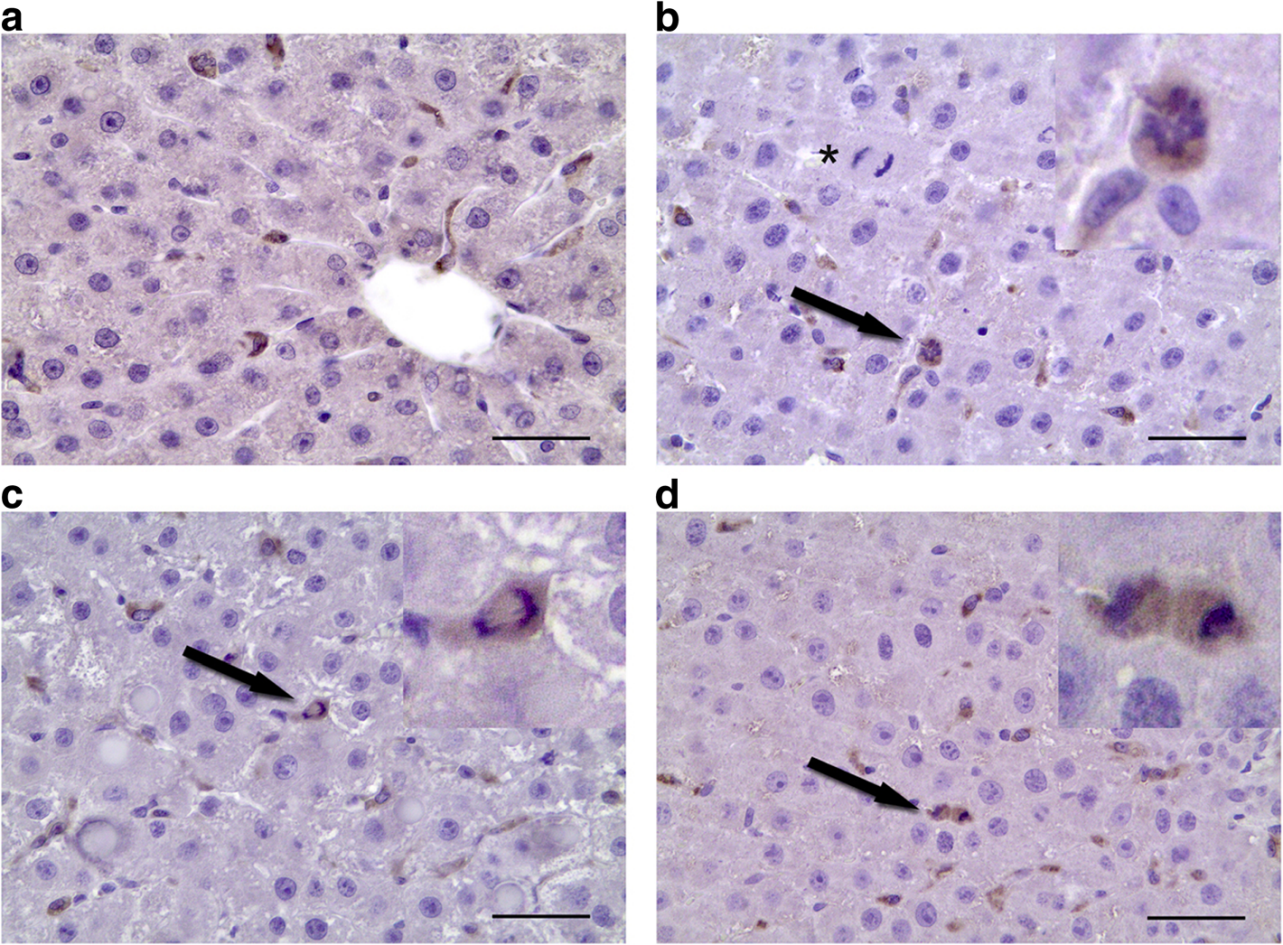
Macrophage proliferation dynamics. CD68^+^ macrophages (brown) in the intact liver (**a**) and in the residual liver at 48 h after the surgery. Arrowheads indicate metaphase (**b**), anaphase (**c**) and telophase (**d**). Bars, 50 μm; cell nuclei are counterstained with hematoxylin (blue). The asterisk indicates a dividing hepatocyte

No mitotically active macrophages were observed in both intact (Fig. 4a) and regenerating hepatic tissue for the first two days after the surgery. Dividing CD68^+^ cells were detected at 48 h after the surgery (Fig. 4b, c and d), with the mitotic index of 4.5 ± 1,8‰, and none of the mitotically dividing CD68^+^ cells were detected after this time point until the end of observation.

### Tnfα protein expression dynamics

**Fig. 5:**
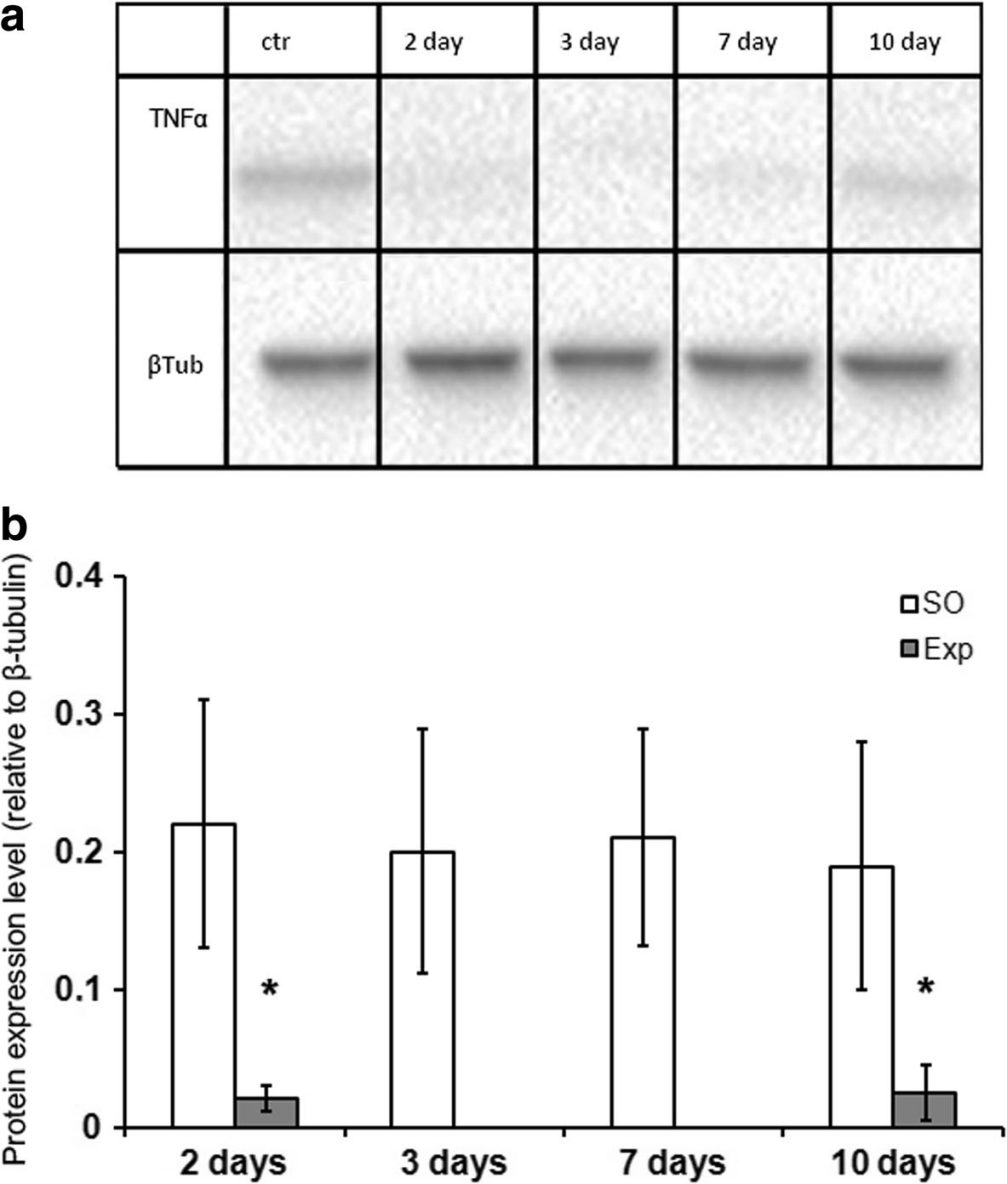
TNFα protein expression in the residual liver during the recovery. Visual assessment of the western blot (**a**) was followed by quantitative densitometry (**b**). SO - sham operated animals, Exp - operated animals. The grouping of blots cropped from different gels. Full-length blots are presented in Additional file 1: Figure 1. The data are represented as mean values ± SD with asterisks indicating statistical significance of the differences (as compared to the control; *p* < 0.05)

As revealed by western-blot analysis, the surgery induced a sharp decrease in Tnfα protein expression within the residual hepatic tissue, and no detectable amounts of this protein were present in the regenerating livers on days 3 and 7 after the surgery (Fig. 5a, and b Additional file 1).

## Discussion

We have previously shown that the late onset of hepatocyte proliferation is characteristic of recovery after SH, as compared with the liver resections of smaller volume. [12]. The first mitotically dividing hepatocytes appear as late as at about 30 h after SH, and this delay has been additionally confirmed in the current study by means of Ki67 immunostaining. After partial hepatectomies of various extent (resections of 30–70% of the organ) the proliferation of hepatocytes invariably begins at 12 h after the surgery [13]. It has been assumed that the delay, associated with the subtotal volume of ectomized hepatic tissue, is caused by prolongation of the arrests at both the G0-to-G1 transition and the exit from G2 [7]. However, the results of Ki67 immunostaining indicate the prevalence of the G0-to-G1 arrest, since no hepatocytes expressing Ki67 are observed at 24 h after the surgery (Fig. 1b). Moreover, no signs of cell cycle arrest at the exit from G2 at the early stages of regeneration are evident, as the increases in the number of Ki67^+^ hepatocytes at these stages are invariably accompanied by increases in the mitotic index of hepatocytes [12] (Fig. 1d, and g). The G2-type of cell cycle arrest, also present in this system, is probably more characteristic of the later stages, e.g. day 5, when the presence of high numbers of Ki67^+^ hepatocytes is accompanied by a significant decrease in the mitotic index ([12], Fig. 1d, and g).

The delay in the onset of proliferation may have several causes. One of them is probably related to a decrease in production of cytokines Tnfα and Il6, which stimulate the entry of hepatocytes into mitotic cycle, coinciding with low levels of Hgf, which is the main mitogen for hepatocytes [14, 15]. These effects could be supported by increased synthesis of Tgfβ, which reportedly inhibits hepatocyte proliferation [3]. Although no apparent increase in *Tgfb1* gene expression is observed within the liver after subtotal resection, the delayed onset of *Hgf* gene expression and the slow recovery of Hgf stocks in the regenerating liver have been reported previously [12].

Significant increases in the *Il6* gene expression, but no increase in the expression of its agonist *Tnfα*, have been detected at 3 and 6 h after the subtotal resection [12]. On days 2, 3, and 7 after the surgery the Tnfα protein levels in hepatic tissue are significantly decreased (Fig. 5, Additional file 1). Both Il6 and Tnfα are known to participate in the initiation of hepatocyte proliferation [3]. It is plausible that the low levels of Tnfα protein in residual hepatic tissue of the rats after SH are related to the prolongation of cell cycle arrest in hepatocytes until 30 h after the surgery. Levels of Tnfα within the liver are provided chiefly by macrophages. The surgery causes activation of the hepatic macrophage system, as manifested by an increase in the macrophage-specific cytokine gene expression and also an increase in the density of macrophages in the residual hepatic tissue. Judging by the negligible expression of CX1CR3 and CD11b markers, the entire population of rat liver macrophages, even during the recovery, is represented by resident Kupffer cells. Thus, the density of liver macrophages during the recovery is increased solely by means of Kupffer cell proliferation, which reaches its maximum at 48 h after the surgery (Fig. 4).

It is still hard to explain why any given mammalian organ or tissue tends to contain macrophages of a single origin, i.e. totally derived from either embryonic hemopoietic sources, or from the monocytes of blood. This probably reflects a subtle difference in functionalities of macrophages derived from these two sources. The monocyte-derived macrophages are typically detected within the liver during recovery from acute toxic damage, e.g. induced by hepatotoxic substances (paracetamol, tetrachloromethane), accompanied by strong inflammatory response [16]. It is evident that Kupffer cells are not capable of full-fledged participation in inflammatory reactions. They probably also have restricted capabilities of stimulating hepatocyte proliferation, which is IL6/TNFα dependent.

The exceedingly large-scale proliferation of hepatocytes, which is required to compensate for the loss of the extremely large volume of parenchyma (80% of the liver mass), should be supported by extraordinary high concentrations of cytokines, including Tnfα, which the resident liver macrophages are unable to provide on their own. The observed proliferation of Kupffer cells may, therefore, not only provide the means for restoration of numerical proportions between hepatocytes and macrophages, but also mitigate the inhibiting effect of Tnfα deficiency.

Specific functional properties of the resident liver macrophages are indicated by characteristic changes in the expression of particular genes, which are known to be specifically expressed in macrophages. The early postoperative period is characterized by elevated expression of both M1 and M2 macrophage-specific genes (respectively, Il1/Il6 and Il10) [12]. Remarkably, the majority of liver macrophages at these stages express CD206 surface antigen, which is a marker of ‘pro-regeneratory’ M2 macrophages. It may turn out that the resident liver macrophages are incapable of polarization to either M1 or M2 direction. If true, this will add to the distinction between them and the bone marrow-derived macrophages and reflect their limited functional abilities.

Any morphogenetic tensions associated with the local macrophage incapability could be possibly solved by immigration of monocytes or monocyte-derived macrophages into residual liver tissue immediately after the resection, but such immigration has been never observed. It is probably prevented by low MCP-1 concentrations insufficient to attract macrophages of bone marrow origin [16]. Deficiency in cytokines and growth factors needed to stimulate hepatocyte proliferation is partly counterbalanced by their synthesis elsewhere. For example, *Tnf* expression in the lungs is upregulated at 30 h after the surgery, simultaneously with the onset of hepatocyte proliferation in the liver [17].

## Conclusions

The results show that the delayed onset of hepatocyte proliferation can be related to low levels of Tnfα in the residual hepatic tissue. The delay is possibly related to specific composition of the liver macrophage population, which is entirely composed of Kupffer cells of embryonic ancestry. These cells express the ‘proregeneratory’ M2 macrophage-specific marker CD206 in the course of regeneration.

## Additional file

Additional file 1: TNFα protein expression in the residual liver during the recovery. SO - sham operated animals, EXP - operated animals. (JPG 458 kb)

## Abbreviations

ALT: Alanine aminotransferase
DAPI: (4’,6-diamidino-2-phenylindole)
IL1: Interleukin 1
IL10: Interleukin 10
IL6: Interleukin 6
TNFα: Tumor necrosis factor α
