# Supplementary figures and images for: Dynamics of macrophage populations of the liver after subtotal hepatectomy in rats
Source: BMC Immunol. 2018 Jul 9;19:23. doi: 10.1186/s12865-018-0260-1 (PMC6038314; doi:10.1186/s12865-018-0260-1)

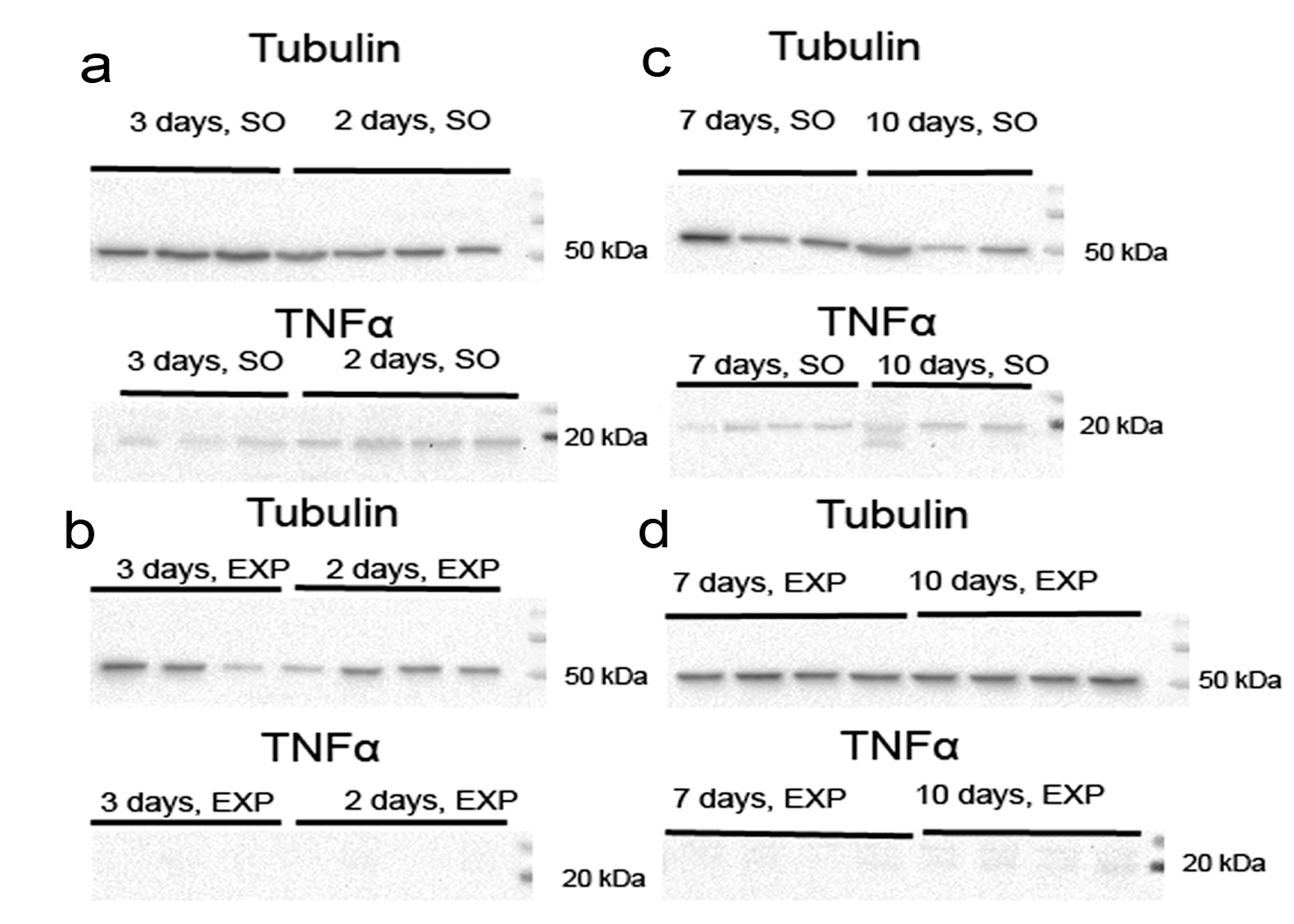

Supplement: Supplementary file 1 — TNFα protein expression in the residual liver during the recovery. SO - sham operated animals, EXP - operated animals. (JPG 458 kb) [file 12865_2018_260_MOESM1_ESM.jpg]
